# Maximizing the performance of protein-based fluorescent biosensors

**DOI:** 10.1042/BST20221413

**Published:** 2023-07-11

**Authors:** Fu Chai, Dazhou Cheng, Yusuke Nasu, Takuya Terai, Robert E. Campbell

**Affiliations:** 1 Department of Chemistry, Graduate School of Science, The University of Tokyo, Tokyo 113-0033, Japan; 2 PRESTO, Japan Science and Technology Agency, Chiyoda-ku, Tokyo 102-0075, Japan; 3 Department of Chemistry, University of Alberta, Edmonton, Alberta T6G 2G2, Canada

**Keywords:** biosensors, chemical biology, fluorescence microscopy, fluorescent proteins, protein engineering, signaling

## Abstract

Fluorescent protein (FP)-based biosensors are genetically encoded tools that enable the imaging of biological processes in the context of cells, tissues, or live animals. Though widely used in biological research, practically all existing biosensors are far from ideal in terms of their performance, properties, and applicability for multiplexed imaging. These limitations have inspired researchers to explore an increasing number of innovative and creative ways to improve and maximize biosensor performance. Such strategies include new molecular biology methods to develop promising biosensor prototypes, high throughput microfluidics-based directed evolution screening strategies, and improved ways to perform multiplexed imaging. Yet another approach is to effectively replace components of biosensors with self-labeling proteins, such as HaloTag, that enable the biocompatible incorporation of synthetic fluorophores or other ligands in cells or tissues. This mini-review will summarize and highlight recent innovations and strategies for enhancing the performance of FP-based biosensors for multiplexed imaging to advance the frontiers of research.

## Introduction

The 1994 demonstration that the green fluorescent protein (GFP) could be recombinantly and functionally expressed in practically any cell type [1–3] was a ‘singularity event’ in biological fluorescence imaging research. With this demonstration, many of the then-current fluorescent imaging technologies became redundant, and a vast new array of technologies and applications became possible.

One of these new technologies was the creation of blue, cyan, and yellow color variants of GFP [4] which could serve as donor–acceptor pairs suitable for distance-dependent Förster resonance energy transfer (FRET) [5]. FRET was already a well-established principle for creating synthetic indicators, so researchers were quick to exploit the GFP color variants for the construction of FRET-based biosensors that exhibit changes in fluorescence hue in response to a change in a biochemical parameter. The earliest examples were FRET-based constructs that changed their fluorescence in response to protease activity [6,7]. Just one year later, in 1997, came the first reports of FRET-based calcium ion (Ca^2+^) biosensors [8,9].

Another enabling technology was GFP's tolerance of circular permutation and insertion of other proteins, as first reported in 1999 [10,11]. Of particular importance to the scope of this minireview, Baird et al. [10] discovered that GFP had a privileged insertion site at which a conformational change of the inserted protein could cause a change in the GFP fluorescence intensity [12]. Baird et al. inserted calmodulin (CaM) at this privileged site to create the Ca^2+^ biosensor designated camgaroo1, which is the archetype of all single fluorescent protein (FP)-based biosensors. In the decades since, this design has been used time and time again to make biosensors for Ca^2+^ [13–15], neurotransmitters [16–18], metabolites [19], membrane voltage [20], and countless other ions, molecules, and biological activities [12,21].

Despite the tremendous progress and broad impact of single FP-based biosensors, practically all biosensors are far from ideal in terms of their performance and properties. At a minimum, a highly optimized biosensor would have a fluorescence intensity comparable or greater than GFP itself, a turn-on fluorescence intensity change (Δ*F*/*F*_0_, calculated as (*F*_max _− *F*_min_)/*F*_min_) in the range of 10–100, and an affinity for its target that is tuned to the biologically relevant range. Ideally, it would also have excellent photostability, far-red or near-infrared (NIR) fluorescence, and narrow excitation and emission peaks. Needless to say, all of these favorable properties need to be retained when the biosensor is expressed *in vivo*. Arguably, the biosensor proteins that come closest to this ideal are late-generation GCaMP variants, the most recent of which is jGCaMP8 [22], which is the product of more than 20 years of development [14]. Parallel efforts based on the mNeonGreen [23] homolog of GFP have led to a variety of alternative Ca^2+^ biosensors [24,25], the most recent of which is the NEMO series [26].

With the ever accelerating pace of biological research, there is an increasingly pressing demand for a greater variety of highly optimized biosensors. The example of the GCaMP series demonstrates that biosensors can be steadily improved, given sufficient time and resources. However, many researchers are now exploring whether there might be faster and more efficient ways to develop and optimize biosensors with close to ideal properties. This mini-review aims to summarize and highlight some of the recent efforts to accelerate the pace of biosensor development and to apply biosensors for multiplexed imaging.

## Accelerating the pace of FP-based biosensor development

FP-based biosensors are powerful tools for studying cellular physiology with high spatiotemporal resolution in cells, tissues, and whole animals. However, many biosensors exhibit limited fluorescence intensity (*F*) and response (Δ*F*/*F*_0_). Developing biosensors with enhanced fluorescence intensity and biological activity-dependent fluorescence response presents numerous challenges, often requiring laborious optimization and large-scale variant screening. As FP-based biosensors are proteins, the techniques used to develop and improve them fall within the scope of molecular biology and protein engineering. More specifically, the engineering of FP-based biosensors typically involves several rounds of linker optimization followed by whole-gene-directed evolution. In this section, we focus on methods to accelerate biosensor engineering and screening approaches.

Linker optimization is one of the most critical aspects of biosensor engineering. The key linkers are those that connect the sensing element to the FP scaffold. Optimal linkers possess the appropriate length, flexibility, and composition to efficiently convert a target-dependent conformational change of the sensing element into a change in the fluorescence intensity of the FP. As one example, during the development of the STEP (sensor for transiently expressed proteins) biosensor, optimization of the linker between the human Bim peptide and cpGFP improved the Δ*F*/*F*_0_ of the biosensor from 1.0 to 3.4 [27].

**Figure 1.**
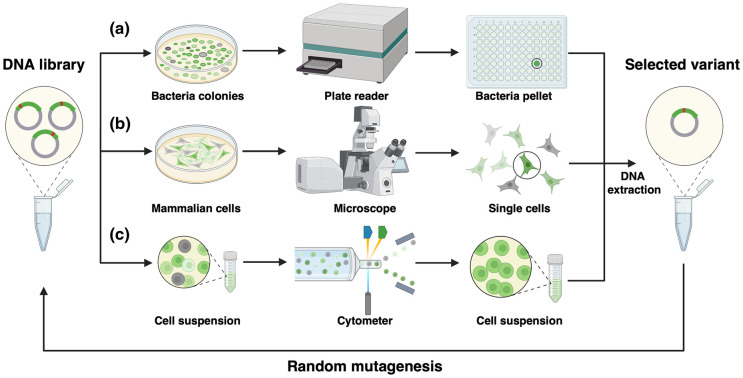
Methods maximizing the performance of FP-based biosensors. (**a**) Whole-gene directed evolution in bacteria. (**b**) Mammalian cell-based screening. (**c**) Microfluidic flow cytometry-based screening.

Within the broad scope of protein engineering, directed evolution is the most powerful technique for improving a protein's properties [28]. Directed evolution involves generating a diverse library of protein variants through mutagenesis or recombination, screening for improved properties using selection methods, and iterating the process with selected variants to further optimize their properties. Since the earliest days of GFP development, researchers have relied on directed evolution [29] to iteratively improve the properties of FPs, and FP-based biosensors. To date, most directed evolution has been performed using bacteria, which offers the greatest amount of versatility and convenience (Figure 1a). Unfortunately, most biosensors are intended for use in eukaryotic cells or in animals, and so there is always a concern about whether the biosensor optimized in bacteria will retain its favorable properties in more complex cell types and tissues.

To increase the probability that a new biosensor will provide satisfactory performance in an animal, it would be ideal to perform the optimization using mammalian or other eukaryotic cells. However, this leads to additional challenges with respect to screening the properties of large libraries of biosensor variants (Figure 1b). In an effort to address this challenge, Rappleye et al. developed a novel mammalian cell-based high-throughput approach, the Optogenetic Microwell Array Screening System (Opto-MASS), which enables functional screening of thousands of variants per day [30]. Similarly, Piatkevich et al. have described a robotic multidimensional directed evolution approach, which combines mammalian cell-based imaging with the isolation of single cells by robotic cell picking [31]. The method is capable of screening 3 × 10^5^ cells expressing different variants in ∼4 h. The utility of this method was demonstrated by using it to develop the opsin-based fluorescent voltage reporter Archon1. Compared with opsin-based voltage reporters previously reported, Archon1 exhibits an excellent localization on neural cell membrane, improved brightness, improved voltage sensitivity to single action potentials, and spectral compatibility with an optogenetic actuator.

While screening by physical separation on plates or in wells often imposes limitations on the number of variants that can be tested, microfluidic cytometry leverages microscale fluid manipulation to enable high-throughput, precise, and real-time measurement of the characteristics of many individual cells (Figure 1c). Lee et al. have reported SPOTlight, a microfluidic cytometry-based method that relies on imaging visual phenotypes by microscopy, precise optical tagging of single target cells, and retrieval of tagged cells by fluorescence-activated cell sorting (FACS) [32]. The authors have demonstrated the effectiveness of this method in the massively parallel screening of single-cell variants for the directed evolution of photostable and bright YFPs. In yet other work, Tian et al. [33] have reported Photopick, a screening platform that enables precise phenotype-activated selection over a large field of view. Photopick has been applied to generate genetically encoded voltage biosensors with improved signal-to-noise ratio and kinetics in cultured neurons and in rodents.

Microfluidic droplet screening is an advanced high-throughput screening method that builds upon microfluidic cytometry. This technique allows encapsulating single cells within discrete, picoliter-sized aqueous droplets to maintain the concentration of cellular analytes, providing enhanced capabilities in parallel processing, compartmentalization, and miniaturization for precise, rapid, and cost-effective cellular analysis. Fiedler et al. [34] have developed a microfluidic droplet platform for the screening and separation of cell populations based on the cellular response of FP-based biosensors upon the addition of an exogenous analyte. Koveal et al. [35] described the BeadScan screening modality, which combines droplet microfluidics and automated fluorescence imaging to significantly accelerate the screening process for FP-based biosensors. This method was successfully used to produce LiLac, a fluorescence lifetime imaging microscopy (FLIM)-optimized lactate biosensor with 1.2 ns lifetime change and a 40% intensity change.

## New approaches for multiplexed imaging with FP-based biosensors

To maximize the information content from a single fluorescence imaging experiment, it is ideal to visualize the readout from multiple biosensors with complementary specificities. However, due to the broad excitation and emission spectra of FPs and FP-based biosensors, the number of biosensors that can be imaged simultaneously, using conventional approaches, is limited to about three or four [36]. There is considerable interest in increasing the number of biosensors that can be assessed at once, thereby enabling the multiplexed measurements of biochemical pathways and processes.

**Figure 2.**
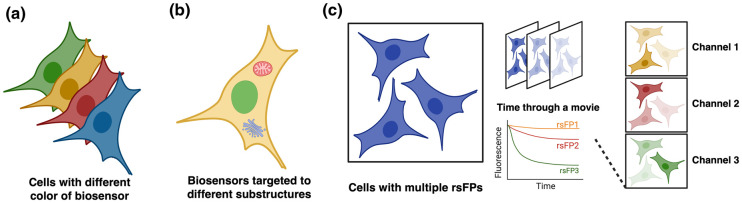
Maximizing the biosensor multiplexing. (**a**) Multicolor imaging. (**b**) Spatial multiplexed imaging targeting to different subcellular compartments. (**c**) Time-resolved temporal multiplexing utilizing different rsFPs.

The most straightforward solution for multiplexing is to employ spectral distinct biosensors that can be easily distinguished based on their fluorescence properties (Figure 2a). Habif et al. [37] reported a triple-modality biosensor system, CASPAM, which expresses three differently colored FP-based biosensors in equimolar concentrations. CASPAM enables simultaneous imaging of the activity dynamics of three key nodes in the caspase network. Numerous attempts have been made to maximize the number of available color channels, with families of red-shifted and blue-shifted sensors engineered to span the visible spectrum. Qian et al. [38,39] developed a series of NIR FP-based Ca^2+^ indicators, NIR-GECO series, which extend the FP-based biosensor color to the NIR range. A spectrally optimized blue-shifted variant of NIR-GECO, iBB-GECO1, was engineered and used with other FP-based biosensors for four-color multiplexed imaging in MIN6 cells and five-color imaging in HEK293T cells [40].

In addition to spectral separation, spatial separation is another approach for the simultaneous imaging of multiple biosensors. To overcome the problem of spectral overlap, biosensors of the same spectral color can be targeted to different locations in the cell (Figure 2b). In a recent example, Werley et al. [41] introduced a technique called MOSAIC, where cells are patterned in arrayed islands, each expressing a FP-based biosensor targeted to a subcellular location. This technique enabled simultaneous recordings of 20 biosensors in parallel in human cells. An alternative approach developed by Linghu et al. [42] targets biosensors to stochastically formed subcellular clusters (SiRIs) to spatially sample the local biological activity. Fusing different biosensors to different pairs of self-assembling peptides causes them to stochastically cluster into puncta that are separated by micrometer distances. In theory, this design could allow any chosen group of existing biosensors to be adapted for multiplexing. In a broader sense, multiplexed imaging could also be achieved across individual cells as well as subcellular compartments. Yang et al. [43] introduced a ‘biosensor barcoding’ technique to enable highly multiplexed imaging of FP-based biosensors. The central concept of this technique involves labeling cells with barcoding proteins composed of fluorophores with unique colors, each targeting a specific subcellular location. The authors demonstrated the simultaneous imaging of up to 72 biosensors.

Temporal multiplexing is an innovative approach that enables multiple signals to be observed simultaneously in cells over extended time frames without interference. Recent progress in this field has focused on fluorescence lifetime imaging technologies and the novel application of temporal properties of FPs. An example of temporally multiplexed imaging is reported by Qian et al. [44], which exploits differences in the reversible photoswitching properties of FPs. In their design, reversibly photoswitchable FPs (rsFPs) exhibiting different behaviors over time during continuous imaging are expressed and traced in cells. By computationally separating the total fluorescence signal at each pixel into a linear weighted blend of the individual reference signals from each fluorophore, the intensity of each fluorophore at a specific pixel can be determined (Figure 2c). Combining spectral and temporal multiplexing enables them to image seven different signals in individual cells which is more than previously possible with FP-based biosensors being imaged in living cells on conventional fluorescence microscopes.

## Expanding the range of biosensor properties using chemigenetic approaches

As discussed above, fully protein-based biosensors are useful tools for addressing many biological questions because they are fully biocompatible, they can be localized to specific cell-types or organelles, and their performance can be substantially improved or customized via directed evolution. However, synthetic fluorophores can have several advantages relative to protein-based fluorophores, such as higher brightness, better photostability, and longer (more red-shifted) wavelength [45,46]. Also, while there are a relatively limited number of suitable protein-based binding domains for metal ions, a wide range of synthetic metal ion chelators have been successfully used to make ion-specific fluorescent sensors [47]. For these reasons, there has recently been a trend towards exploring ‘chemigenetic’ indicators composed of both a protein-based component and a synthetic component [48–50]. The advantage of such chemigenetic designs is that they can, in principle, combine the advantages of both synthetic and protein-based indicators.

The key technology that makes chemigenetic indicators possible are ‘self-labeling’ proteins which bind (typically via the formation of a covalent bond) to appropriately designed small molecule ligands. As the protein part can be targeted by using specific promoters or genetic fusion to organelle marker proteins, researchers can control the localization of organic molecules by simply adding the compound with a corresponding ligand moiety to the tissue under investigation. Examples of self-labeling tag proteins include HaloTag [51], SNAP-tag [52], PYP-tag [53], eDHFR [54], FlAsH and ReAsH systems [55,56], FlARe [57], and FAST [58] tags. Among those systems, SNAP-tag and HaloTag proteins are the prototypical examples of systems in which a covalent bond is formed to a small molecule, while FAST system is a prototypical example of system in which a small molecule non-covalently binds to protein.

HaloTag is a protein engineered from a bacterial haloalkane dehalogenase (DhaA). The wild-type protein catalyzes the dehalogenation of a chloroalkane using a Glu-His-Asp catalytic triad [59]. The reaction mechanism begins with S_N_2 displacement of the substrate halogen by the active-site aspartate residue. The resulting ester intermediate is then hydrolyzed through general-base catalysis, with the assistance of the histidine residue. To engineer HaloTag from DhaA, this histidine was mutated to a phenylalanine that is unable to assist in the hydrolysis step. Accordingly, a stable ester bond is formed between the protein and the dehalogenated substrate. Despite its relatively large size, due to its favorable properties such as rapid labeling kinetics and high stability, HaloTag has been widely used as a self-labeling protein tag for bioimaging. SNAP-tag was developed from human *O*^6^-alkylguanine-DNA alkyltransferase (hAGT) by random mutagenesis and directed evolution [60]. It binds to an *O*^6^-benzylguanine ligand via a nucleophilic addition efficiently but with slower kinetics compared with HaloTag. Nevertheless, the smaller size of SNAP-tag makes it less likely to perturb the function of the protein of interest. Both HaloTag and SNAP-tag have been further optimized and customized, resulting in variants such as the CLIP-tag that reacts with *O*^2^-benzylcytosine derivatives [61], HaloTag8 that avoids the formation of an internal disulfide bond [62], and HaloTag9 with brighter fluorescence when bound to rhodamine derivatives [63].

Relative to covalent labeling systems, non-covalent labeling proteins have the possible advantage of dye exchange, which can lead to less apparent photobleaching during imaging due to the exchange of photobleached dye molecules. One of the key examples of a non-covalent system is Y-FAST which reversibly binds to rhodanine-benzene derivatives, and was engineered from photoactive yellow protein by directed evolution using yeast display and FACS. Recent work has proved that fluorogen renewal can significantly reduce the apparent photobleaching rate of Y-FAST [64]. Another recent example is a HaloTag-based system that is engineered to employ non-covalent binding to ligands. In this system, the ligands are methylsulfonamide or trifluoromethylsulfonamide alkane ligands (rather than chloroalkane ligands) which bind in the active site [65].

**Figure 3.**
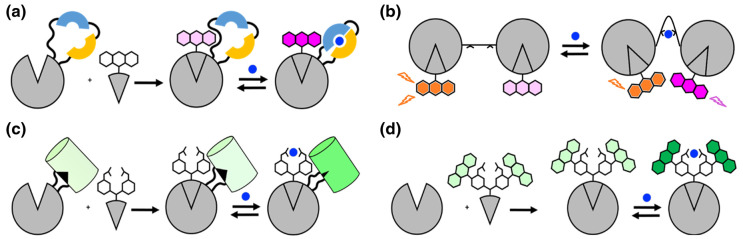
Summary of chemigenetic indicator designs. In all designs, a protein-based component is modified with a synthetic component, potentially combining the advantages of both types of molecules. (**a**) Indicators based on a synthetic fluorophore and protein-based sensing domain (e.g. HaloCaMP [49]). (**b**) FRET-based chemigenetic indicators (e.g. SH-AKAR3EV [71]). (**c**) Indicators based on a synthetic sensing domain and protein-based fluorophore (e.g. HaloGFP-Ca and HaloGFP-Na [50]). (**d**) Indicators based on a synthetic indicator with a ligand moiety and a self-labeling protein (e.g. BOCA-1-BG [72] and TLSHalo [74]).

The chemigenetic indicators reported to date can be classified into four general categories: (1) Protein-based target-binding domain with a single synthetic fluorophore (Figure 3a); (2) Protein-based target-binding domain with two synthetic fluorophores (FRET-based sensors; Figure 3b); (3) Protein-based fluorophore with a synthetic target-binding moiety (Figure 3c); and (4) Protein-based localization of a synthetic indicator (i.e. fluorophore plus target-binding moiety; Figure 3d).

**Figure 4.**
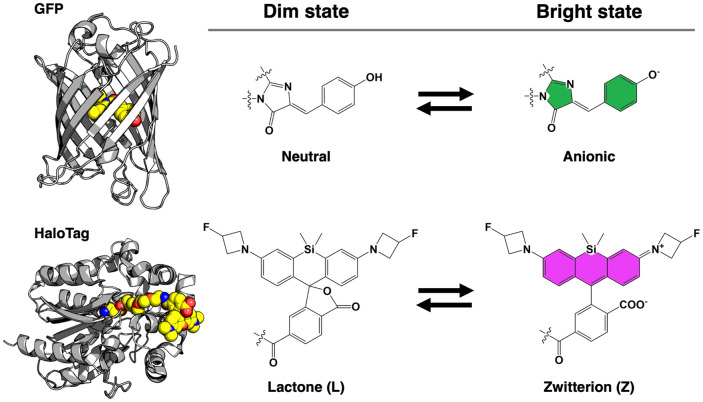
Fluorogenic response mechanism of GFP and a HaloTag-rhodamine conjugate. Equilibria between the dim (or non-fluorescent) state and the brightly fluorescent state, of GFP (PDB ID 1EMA) [79] and a representative HaloTag-rhodamine conjugate (PDB ID 6U32) [49], are shown. The L-Z equilibrium is represented using the structure of JF_635_ [67].

Indicators in the first category are composed of a synthetic fluorophore and protein-based sensing domain, connected by a self-labeling protein. In these indicators, upon binding to the analyte or sensing signal changes, the sensing domain undergoes a conformational change that alters the environment of the synthetic dye, thus causing a fluorescence change (Figure 3a). Rhodamine dye derivatives are the most commonly used dyes for this purpose since they exist in an equilibrium between a non-fluorescent lactone (L) and a fluorescent zwitterion (Z) (Figure 4). Classic rhodamine dyes such as tetramethylrhodamine (TMR) strongly favor the fluorescent zwitterionic form. However, some engineered rhodamine derivatives exist predominantly in the L form in aqueous solution but adopt the Z form when conjugated to a target protein, leading to high fluorogenicity. Such rhodamine dyes have been further optimized to absorb and fluoresce at far-red to NIR region wavelengths, with superior brightness and photostability. A typical example is the Janelia Fluor rhodamine derivative dye (JF dye) series [66,67]. A series of far-red JF dyes has been applied to create a series of chemigenetic indicators, among which notable examples are the HASAP and HArcLight voltage indicators, and the HaloCaMP Ca^2+^ indicator [49]. There are also examples based on the FAST non-covalent binding scaffold, which makes use of the reversible non-covalent binding of the synthetic dyes. The FAST system [68] and the UnaG bilirubin-binding FP system [69] have been used for bimolecular fluorescence complementation and as cell cycle indicators that have much faster responses than fully protein-based ones.

These kinds of chemigenetic systems have been expanded for the development of FRET-based sensors [48,70,71] by utilizing the versatility of spectral range of the synthetic dyes as the second category (Figure 3b). This is realized by introducing a FRET pair of fluorophores, either both synthetic or a pair of synthetic dye and protein-based fluorophore, with different spectral properties into a single system. The examples are a series of kinase indicators, based on chemigenetic SNAP-tag and HaloTag domains combined with JF dye series [71]. A change in distance or orientation between the fluorophores due to the target binding allows the FRET process to take place. A related example is the Voltron chemigenetic voltage indicator that is based on FRET between a JF fluorophore-labeled HaloTag and an opsin protein that undergoes membrane potential dependent changes in absorbance [48]. This type of chemigenetic approach expands the versatility of spectral properties and also provides considerable flexibility in the choice of dye colors to use for a particular experiment.

Indicators in the third category (protein-based fluorophore with a synthetic target-binding moiety) were developed to address the fact that the range of protein-based target-binding domains for metal ions is relatively limited. The sensing protein that binds to the target must also have enough conformational change to change the environment of the fluorophore (either a FP or a synthetic dye). To eschew the limited range of protein-based sensing domains and further expand the range of chemigenetic indicators, our group developed chemigenetic indicators based on the combination of a protein-based fluorophore and synthetic target sensing domains (Figure 3c) [50]. This effort has led to the development of a HaloTag plus GFP fusion protein (HaloGFP) in which the GFP chromophore is positioned close to the HaloTag active site. To create a Ca^2+^ indicator, HaloGFP was treated with a BAPTA HaloTag ligand (HaloGFP-Ca). To create a Na^+^ indicator, HaloGFP was treated with a Na^+^-binding crown ether HaloTag ligand (HaloGFP-Na). Although this class of chemigenetic indicators is still in its infancy, this design may overcome some of the current limitations of protein-based biosensors.

Indicators in the fourth category (protein-based localization of a synthetic indicator) are based on the idea of directing the localization of an existing high-performance synthetic indicator using self-labeling proteins (Figure 3d). Examples include indicators for Ca^2+^ [72,73], K^+^ [74], Na^+^ [75], and Zn^2+^ [76]. However, for these types of indicators, the synthetic ligands attached to tag proteins tend to be very large, therefore, poor membrane permeability and poor biocompatibility can represent major challenges.

## Summary and outlook

In this mini-review, we have aimed to summarize the current state of the art with respect to improving the properties and applications of single FP-based biosensors. The three major directions that we highlighted in this review are: (1) Efforts to accelerate the directed evolution of biosensors; (2) Efforts to maximize the utility of biosensors for multiplexed imaging; and (3) Efforts to improve biosensors using chemigenetic approaches.

Looking forward, we anticipate that the near future will see substantial advances in all three of these directions. In the area of accelerating the directed evolution of biosensors, we expect to see continual improvements in methods for screening ever larger libraries of biosensors in eukaryotic (and ideally mammalian) cells. Though we also suggest that there should be greater attention paid to developing methods that aim to increase the number of cycles of evolution. In our anecdotal experience, a greater number of rounds of evolution with modest library sizes tends to produce better outcomes than smaller numbers of rounds of evolution with larger library sizes. Taking this idea to the logical extreme, we might one day expect to see the continuous directed evolution of genetically encoded biosensors. Phage-assisted continuous evolution (PACE) in bacteria is a method for evolving protein-protein interactions in a continuous fashion [77]. It is not obvious how such a system could be applied to FP-based biosensors, but perhaps an ingenious researcher can adapt a PACE-like system for the rapid evolution of biosensors with significantly improved properties and performance.

In the area of multiplexed imaging of biosensors, we expect to see a continual increase in the number of biosensors that can be imaged in live cells. Currently, there remains a large gap between the number of biosensors that can be imaged in live individual cells (using spectral or spatial separation, for example), and the number that can be imaged in separate co-cultured cells or fixed cells (using spectral or molecular barcoding, for example). We anticipate that the most impactful developments in this area will come from increasing the number of biosensors that can be imaged in individual live cells (ideally *in vivo*) in real-time. To achieve this, we expect that techniques that combine spectral and spatial separation with other spectral signatures such as fluorescence lifetime, Raman signatures, or fluorescence fluctuations, might be particularly fruitful.

In the area of chemigenetic biosensors, we anticipate that the most important future direction will be the development of improved fluorescent dye molecules for *in vivo* imaging applications. The ideal fluorescent dye would be NIR fluorescent, highly bioavailable, highly fluorogenic upon interacting with the target protein, highly photostable, and minimally phototoxic. It remains unclear if it is possible to design and synthesize such a fluorophore with this combination of ideal properties, since some properties (e.g. bioavailability and fluorogenicity) seem to be mutually exclusive [78]. Other properties may by inherently correlated. For example, decreased phototoxicity is likely to be an accompanying benefit of using a dye with a more red-shifted (that is, lower energy) excitation peak. Overall, we are hopeful that a chemist's intuition, or well-trained machine-learning algorithms, will lead to the development of such an ideal fluorophore.

In conclusion, it is an exciting time in the field of biosensor development, with breakthroughs occurring frequently on multiple fronts. While we have dealt with three major directions separately in this mini-review, there is of course a high degree of synergy between these directions. New methods for accelerating biosensor development are as applicable to chemigenetic biosensors as they are to fully genetically encoded sensors, and both types of sensors can be used in conjunction to maximize multiplexed imaging. Together, these technological advances will continue to push the frontiers of biological research.

## Perspectives

- FP-based genetically encoded biosensors have revolutionized the ability of researchers to achieve multiplexed visualization of biochemical processes as they occur in cells, tissues, and animals.
- The traditional methods for developing FP-based biosensors have been tremendously successful, but they are also time and labor intensive. Researchers have recognized the need for new methods to rapidly and effectively produce high-performance biosensors.
- Advances in methods for the directed evolution of FP-based biosensors, new approaches for multiplexing of biosensors, and new strategies for chemigenetic augmentation of biosensor function, are accelerating biosensor development and applications.

## Open Access

Open access for this article was enabled by the participation of University of Tokyo in an all-inclusive *Read & Publish* agreement with Portland Press and the Biochemical Society under a transformative agreement with Individual.

## Abbreviations

CaM: calmodulin
DhaA: haloalkane dehalogenase
FACS: fluorescence-activated cell sorting
FP: fluorescent protein
FRET: Förster resonance energy transfer
GFP: green fluorescent protein
NIR: near-infrared
